# A Treatise on the Diseases of the Brain and Spinal Chord in Children, Illustrated by Cases Occurring in the Wards of the First Children's Hospital

**Published:** 1846-04

**Authors:** 


### Art. V.

*Die Krankheiten des Gehirns und Rückenmarks bei Kindern. Durch Krankheitsfälle aus dem ersten Kinderspitale erläutert, von* Dr. L. W. Mauthner.—*Wien,* 1844.

*A Treatise on the Diseases of the Brain and Spinal Cord in Children, illustrated by Cases occurring in the Wards of the First Children's Hospital.* By Dr. W. L. Mauthner.—*Vienna,* 1844. 8vo, pp. 456. With five lithographed plates.

The profession is much indebted to Rokitansky and other members of the modern Vienna School for their zealous cultivation of pathological anatomy. Practical medicine, however, is a department in which they have of late laboured but little, or to little purpose, if we except the physical diagnosis of diseases of the chest, which Professor Skoda and his followers have done so much to elucidate. This being the case, there is some danger of our over estimating the value of a work in which the author's aim has been entirely practical: just as the rarity of a gift always seems to enhance its worth.

The merits of Dr. Mauthner's work, however, are such as would secure for it attention whenever it might have appeared. It contains the results of seven years' careful observation in the wards and among the out-patients of the children's hospital which he established in the year 1837, and which has since flourished under the patronage of the empress. The hospital contains 36 beds, and between 2000 and 3000 children are admitted as out-patients, some of whom are visited at their own homes. Of 15,836 children who have thus come under his notice, 1747, or about 11 per cent., were suffering from some disorder of the cerebral system, of whom about 10½ per cent. died. The frequency and the importance of diseases of the brain and spinal cord in childhood have induced Dr. Mauthner to make them the subject of this volume; and he promises at some future time to investigate in a similar way the other diseases of early life.

"Diagrams," says the author, "are in geometry just what the histories of cases are in medicine. The former illustrate the proposition, and serve to reduce it to absolute certainty; the latter illustrate the laws which regulate nature even when suffering, and lead to as complete a measure of clear perception as is to be attained in an experimental science."

The whole book is a kind of practical commentary on this motto; for it contains the minute details of 123 cases, connected by general observations on the diseases of which they are instances. Such an arrangement unquestionably presents many advantages, but inseparable from it are several inconveniences which greatly detract from its usefulness. No clear description is given of any affection, but its peculiarities have to be learnt by the somewhat tedious process of reading several cases. The particulars of one case suggest remarks on one striking character of the disease, and, eight or ten pages farther on, another case suggests the addition of a new feature to the portrait. It thus happens that no adequate conception is formed of the subject as a whole, from its not being represented, except in parts. Another defect, traceable to the same cause, is, that the author's remarks are too often merely suggestive, and his descriptions so brief, that, though their truthfulness would at once be recognized by those who are already familiar with their subjects, they are frequently little more than allusions which would be wholly inadequate to teach the inexperienced. But, though these circumstances somewhat limit the utility of this work, it will yet fully repay an attentive perusal, since it contains very much practical information, gathered, not from the writings of others, but from personal observation at the bed-side of the sick.

The book commences with some remarks on the frequency of diseases of the cerebral system, on their diagnosis and treatment. The author then passes to the consideration of the special diseases of the brain, which he has arranged under the following heads: "1, Congestion, irritation of the brain (*febris cephalica*); 2, Apoplexy; 3, Inflammation of the brain (*encephalitis* and *meningitis*); 4, Acute hydrocephalus; 5, Hypertrophy of the brain; 6, Atrophy of the brain; 7, Chronic hydrocephalus; 8, Tubercle of the brain; 9, Softening of the brain; 10, Cerebral convulsions." (p. 12.)

Cerebral congestion. This, though not in strict propriety an independent disease, is yet so frequent an attendant on the different maladies of childhood, as to merit a separate examination of its causes and symptoms. Its frequency is so great, that Dr. Mauthner observed it in 186 out of 229 children who had died of various diseases. In a large proportion of these cases it occurred in all probability in the very article of death, which tends to empty the arterial and gorge the venous vessels throughout the whole system. In the course of various diseases, however, and under various circumstances, the brain may become overloaded with blood, as the result of an exaggerated or an enfeebled activity in the cerebral vessels and the vascular system in general. On this difference of cause the author founds a distinction of cerebral congestion into the active and the passive, either of which varieties may be acute or chronic. He further attempts a distinction between that form of cerebral congestion in which the surface of the brain is the seat of the affection, and that in which the central parts of the organ are chiefly involved. He confesses, however, that this distinction is no longer practicable when a state of intense congestion of any part of the brain is present; and we have much doubt whether the differences he indicates at p. 14 do not depend on the degree of the affection and the suddenness of the seizure more than on the seat of the congestion.

*Febris cephalica.* Intermediate, between the acute form of active congestion and the chronic form, Dr. Mauthner places what he calls the *febris cephalica,* which is attended by fever, with pain in the head, disorder, or, more generally, constipation of the bowels, and a train of symptoms, such as are often supposed to be premonitory of an attack of acute hydrocephalus. Dr. Mauthner is of opinion that it sometimes occurs as an independent affection, when it tends to a critical termination by sweat and urine on the third or fifth day. We cannot quite subscribe to the correctness of this last observation, for we do not think that there is any form of cerebral congestion so distinctly separated from others by its symptoms and by its tendency to critical termination at a certain period as to need a distinct place in our nosologies.

It is only in the most active forms of cerebral congestion, and in children above six months old, that the author attempts general depletion, and he adds (at p. 17) a very useful caution against inferring anything as to the propriety of venesection from the excitable pulse, and the changeable hue and temperature of the surface in young children. In milder cases less active treatment may suffice, as the employment of cold applications to the head, the administration of purgatives, of small doses of calomel, and of antiphlogistic remedies, which it is of great importance to administer in a form as nearly as possible tasteless.

The writer defines "passive congestion" as "that form of morbid accumulation of blood in the brain which takes place without violent symptoms of reaction and irritation." (p. 27.) In new-born children who breathe but imperfectly, and whose blood circulates languidly through the brain, this condition is physiological. If, however, there should be much delay in the establishment of respiration, a condition ensues of sopor, heaviness of the head, and want of power over the extremities; the heart beats irregularly, and convulsive twitchings occur. The state of passive congestion kept up by this imperfect respiration gives rise, in the course of time, to serous effusion, and this effusion may become very considerable without inducing reaction in the vascular or nervous systems. By slow degrees the cerebral substance becomes softened, partly by imbibition of the effused serum, partly by the serum being forced out into the parenchyma of the brain, and death ensues from direct paralysis of the nervous centre. This is the condition described by Billard as non-inflammatory softening of the brain.

In older children passive congestion of the brain is a frequent result of various diseases, both acute and chronic, which give rise to much exhaustion of the nervous power. It is likewise by no means unusual in children who have been exposed to unfavorable hygienic conditions, or who are ill fed. It is also readily induced by the over energetic treatment of active congestion, and the abstraction of too large a quantity of blood. The symptoms which attend it are very different from those of active congestion:

> "They commonly consist in a pale and cool, or hot, though moist skin, eyes sunken in the head, and half closed, drowsiness without sleep, weariness, heaviness of the head, stupor, diminished sensibility, impaired digestion; and hence vomiting and purging of food, for the most part indigested, small and frequent pulse, and swelling of the veins of the head. To these must be added sopor and convulsions in the severer degrees of the affection." (p. 31.)

The treatment of this state consists in the use of warm mustard baths, and in the employment of mild diaphoretics and aperients. Alteratives, and even emetics, which latter are quite contraindicated in active congestion, are sometimes of great service, nor is the use of narcotics absolutely excluded. After mentioning a few other points that deserve attention in the management of this condition, and relating several illustrative cases, the author passes (at p. 40) to the next subject of his treatise.

APOPLEXY. He notices the rarity of circumscribed effusions of blood into the brain in infancy, although the symptoms of apoplexy are by no means uncommon. A state of general plethora even is not always essential to the production of apoplectic symptoms, but they may occur from general weakness and consequent diminution of the propulsive power which should impel the blood through the brain. Interrupted circulation, indeed, however produced, may produce apoplexy in early life—a fact, of which the peculiarities in the structure of the infantile brain and skull afford a satisfactory explanation.

> "The impulse of the heart is powerfully felt in the cerebral vessels, while neither the external counter-pressure, nor the tension of the surrounding parenchyma is such as to assist its reflux. In proportion to the rapidity with which the atmospheric pressure propels the blood inwards, will be its accumulation in the long, fine, and numerous cerebral vessels, which have no firmer support than the soft tissue of the brain. So soon therefore as by any acceleration of the blood's motion the carotids bring a relatively larger amount of blood to the brain than the jugulars can carry off, danger of apoplexy arises." (p. 41.)

*Cephalhæmatoma.* Those very peculiarities, however, which tend to favour the accumulation of blood in the brain, either by increased afflux, or by diminished or interrupted efflux, are associated with another which serves the part of a safety-valve, and provides an outlet for its excess. This consists in the very free communications of the cerebral vessels with those of the cranium and the scalp. Hence, in early childhood, effusions of blood beneath the scalp are frequently met with, constituting the *apoplexia tegumentaris* of Billard, and cephalhæmatoma is regarded by the author, in common with Rilliet and Barthez, as the result of causes that tend to produce sudden and violent stasis of the blood in the brain, and as another instance of the utility of this safety-valve arrangement.

In older children, in whom these communications have, with the progress of ossification, become less numerous, death from apoplexy without any extravasation of blood is not very unusual. Such an instance is case 15, in which a healthy boy, aged 5 years, after overloading his stomach with indigestible food, was suddenly seized with convulsions, became livid and comatose, and died in thirty-eight hours. The brain was gorged with blood, but no vessel was lacerated, and no exudation of blood had taken place. The converse of this holds good also, and hemorrhage may occur without giving rise to any symptom of apoplexy, of which case 20 is a striking example.

*Passive apoplexy.* Some of the cases most difficult of explanation, are those of what Dr. Mauthner terms passive apoplexy, in which apoplectic symptoms occur in children who are neither plethoric nor possessed of vigour of constitution. He adopts much the same explanation of their occurrence as has been suggested by Hachmann with regard to the same condition which he has described under the name of *apoplexia venosa.*

"It is a well known fact," says the author, "established by Magendie's ingenious experiments, that a very slight deviation from the normal properties of the blood suffices to impede its free circulation through the minuter capillaries. No alteration of the blood seems to arrest its passage through the finer vessels to so great a degree as that which depends on a deficiency of oxygen, and a predominance of alkaline constituents. While the circulation is thus retarded imbibition of the blood by the coats of the vessels goes on, the fluid becomes infiltrated into the neighbouring tissues, whence stasis of the circulation, œdema, and even inflammatory action may result. These results of deficient oxygenation of the blood take place most rapidly, when the mucous membrane of the intestinal canal has become diseased, (under the influence of conditions unfavorable to health, especially if with these there be associated a warm, damp, and foggy state of the atmosphere,) and excretes a large quantity of matter abounding in oxygen, the waste of which, owing to the progressive weakening of the respiratory function, becomes every minute more and more imperfectly supplied." (pp. 62-3.)

Inflammation of the Brain. Dr. Mauthner endeavours to distinguish between inflammation of the brain or its membranes, and acute hydrocephalus; since he thinks, and with justice, that the collection of serum in the ventricles is neither the only indication of inflammation of the brain, nor indeed an invariable attendant on inflammatory processes. He states that he met with fluid in the ventricles in 172 out of 229 post-mortem examinations of children who had died of various affections, and that in 123 instances the fluid present was in considerable quantities.

"Encephalitis," says he, "is an independent, inflammatory process, either completely idiopathic, or occurring as a secondary result of some of the dyscrasiæ; hydrocephalus acutus, on the other hand, is the local manifestation in the brain of various morbid processes: as typhus, scrofula, impetigo, softening of the brain, scurvy, etc. A stage of inflammatory reaction may occur as one among the group of symptoms that attend this affection, but this does not, as in the case of encephalitis, constitute the disease itself, since hydrocephalus may occur, as what is called the *waterstroke,* without any previous stage of inflammation. Acute hydrocephalus, therefore, is always a secondary affection, while encephalitis often occurs as a primary disease, although it may likewise be excited by the existence of some other disease in the system. Acute hydrocephalus never occurs as the consequence of external influences alone." (pp. 76-7.)

There is great similarity between the two affections, so great indeed as to make the attempt to distinguish between them appear at first sight a refinement of little use. There is, it must be allowed, no single symptom that can be mentioned as pathognomonic of either, but the different order in which the symptoms occur constitute, as the writer has well pointed out, the grounds of diagnosis. The stupor and unconsciousness which often exist at the commencement of encephalitis, usually occur towards the end of hydrocephalus. The former runs a rapid course, the latter advances slowly, passing through several stages, often presenting distinct intermissions. Rapid and causeless emaciation mark the commencement of hydrocephalus, and death is often unattended by convulsions; emaciation occurs later in inflammation of the brain, and convulsions are never absent. Another point of difference is found in the age of the subjects who are attacked by the two diseases; hydrocephalus seldom occurring before the end of the first year, and being most frequent from the age of 2 to 7, while inflammation of the brain may come on at any period.

*Hydrocephalus.* In these remarks Dr. Mauthner has, we think, seized very accurately the distinguishing marks which characterize two forms of disease frequent in early life. The one, acute in its course, tending rapidly to a fatal result if left to itself, but amenable to medical treatment; the other, a secondary affection, slow in its advance, often insidious in its progress, but uninfluenced by remedies. The former of these affections is manifestly inflammatory in its nature, of the essence of the other we know but little, but are wont to characterize the assemblage of symptoms which betoken its existence by the name of hydrocephalus acutus. What then is this formidable disease, and wherein does it consist? Dr. Mauthner's definition we confess does not satisfy us, we almost think it does not satisfy himself.

"The name of acute hydrocephalus," says Dr. Mauthner, "is to be applied to every morbid collection of serum in the ventricles, which supervenes in the course of other general diseases, is occasioned (*bedingt*) by them, and runs an acute course." (p. 107.)

We cannot but regard this definition as an advance in the wrong direction, and as tending to involve in hopeless confusion a subject already sufficiently obscure. We know that the symptoms of what is called acute hydrocephalus by no means invariably depend on the effusion of serum into the lateral ventricles, while most extensive collections of fluid are found after death where during life no symptoms have betrayed their existence. Thus in three fourths of all phthisical patients whom M. Louis examined after death, he found extensive effusion into the ventricles. Dr. Mauthner, himself, allows (at p. 291), that the symptoms of tubercular meningitis are, in the greater number of instances, only a repetition of those which constitute acute hydrocephalus, yet in this form of disease, serous effusion is not invariable in its occurrence, and when present is certainly by no means the most important of the post-mortem appearances. Had the author submitted his numerous observations to a rigid analysis, he would, we are sure, have given us something much clearer and more definite as the results of his labours.

*Hypertrophy of the brain.* We are glad to pass from the vagueness which characterizes this part of his subject to another form of disease to which the author has devoted much attention, and has treated of rather fully. It has long been a well-known fact that the brain may, like other organs of the body, exceed its natural size, and that this hypertrophy of its substance is generally associated with some derangement of its function. Few observers, however, have devoted much time or trouble to the study of this morbid condition, or of the symptoms to which it gives rise, a circumstance which imparts a double interest to the results of M. Mauthner's careful investigations.

M. Mauthner has been at the pains of weighing the brain of 216 children at all ages from birth up to the 8th year, during the whole of which period an increase in its weight is pretty constantly going on.

"During this time," says he, "we find a minimum of oz. 10. ʒvj rise to a maximum of oz. 44½. The average weight begins with 13½, and rises to 35½ ounces. During the first year it grows from 13½ to 20½, or 7 ounces; in the second, from 20½ to 25½, or 5 ounces; in the third, from 25½ to 32, or 6½ ounces; and between the fourth and eighth year, from 32 to 35½, or 3½ ounces. Hence it appears that the brain grows most rapidly in the first year of life, that in the second and third years its increase is still considerable, but that its growth is slower after the fourth year. In conclusion it may be observed, as a remarkable fact, that the minimum weight usually occurs in cases of atrophy or phthisis, the maximum in pneumonia, scarlet fever, apoplexy, and cerebral tubercle." (p. 162.)

It further appears, from a minute examination of the condition of the brain in these cases, that its weight is to a great degree dependent on the quantity of blood which it contains.

The general anatomical characteristics of hypertrophy of the brain need no special description, we may notice, however, the author's mention of its frequent coincidence with enlargements of the thymus gland, of the left ventricle of the heart, and of the liver; facts which lend some support to Münchmeyer's theory of the connexion of asthma thymicum with hypertrophy of the brain *. Besides the more usual form of cerebral hypertrophy, in which there is actual increase of the size of the brain, there is another variety in which the increase is only relative. In such cases the brain is not too large but the skull, probably as the result of excessive activity of the process of ossification is too small, and the brain, in consequence of the pressure that it undergoes, acquires a degree of firmness amounting to induration. In such cases the child is always deficient in intellectual power, and is frequently idiotic and unable to walk. The head retains its natural size, but the sutures close unusually early, and the parietal and occipital protuberances are unusually prominent. Such children present none of those indications of rachitis which so often coincide with hypertrophy of the brain, but the lower animal life thrives at the expense of the higher; the skin is firm, and the body fat and ruddy, the muscles and bones strong, the constitution robust, and appetite craving.

A very interesting case is related, at p. 184, in which the symptoms of hypertrophy of the brain depended neither on enlargement of that organ, nor on preternatural smallness of the skull, but on thickening of its diploe as the consequence of rickets.

"The bones of the skull were extremely vascular, the diploe was spongy, an inch thick anteriorly, somewhat thinner posteriorly, but its thickness was everywhere greater than natural. Both the anterior and posterior fontanelles were

* See his essay on the subject in the Zeitschrift für die gesammte Medicin, 1842. Nov. 3.

open; at the posterior inferior angle of the right parietal bone were two spots, at which the walls of the bone were exceedingly thin; the vitreous table presented remarkably deep impressions of the cerebral convolutions, as well as channels for the vessels, particularly on the right side. The dura mater was firmly adherent, the pia mater injected, the convolutions of the brain very distinct, the brain firm, small, all its parts indurated, and weighing 24 ounces." (p. 185.)

It would not be possible during life to form a clear diagnosis of such a case; we mention it rather as a pathological curiosity.

In detailing the symptoms that ordinarily attend hypertrophy of the brain, M. Mauthner distinguishes the passive from the active form of the affection. In passive hypertrophy the cranium early presents a striking deviation from its natural appearance, in the enlargement and globular prominence of the occiput. The parietal protuberances subsequently project, the coronal and sagital sutures continue open in the ninth, or even in the twelfth month, and the fontanelles remain unclosed for a much longer time than natural; the growth of hair is scanty, and the veins of the scalp are much injected. Children in this state sleep much, though they are easily startled, they sweat much about the head, and when in a sitting posture the head drops forward by its own weight. Attacks of crowing inspiration occur when the child cries, and not unfrequently end in, or are accompanied by regular convulsions, and the severity and frequency of these seizures are greatest during the period of dentition. Digestion is at the same time impaired, and vomiting and diarrhea are frequent. By degrees the symptoms of pressure on the brain become more evident, or they are suddenly developed as the result of the supervention of some other disease.

"When hypertrophy of the brain has reached this stage, the skull deviates still more from its natural shape, the forehead sometimes becomes prominent and globose like the occiput, and while the skull goes on acquiring an increased curvature, the region of the temples continues flat and thus contributes to give to the head the appearance of being formed by the union of segments of four spheres. During this stage of the affection the preternatural softening and thinning of the cranial bones, corresponding to the prominences of the convolutions are distinctly perceptible, especially at the occiput. The functions of the brain become now much disturbed, headache, giddiness, impairment of muscular power, and loss of memory occur, the child grows sullen, peevish, sleepless, whimpers continually, and rolls the head constantly from side to side. At the same time it seems choked with phlegm, while the skin becomes every day more flabby, the muscles shrink, the bones grow soft, and the muscular power rapidly diminishes. Hence these children lie usually on their back, breathing with habitual wheezing, and suffering from constant dyspnœa, with occasional asthmatic seizures, such as have been already described. When in this condition slight causes suffice to produce a general excitement of the vascular system, and to excite diseased action in other parts, which render still more obvious the influence of the hypertrophy on the nervous system generally. If the child happen to catch a slight cold, attacks of convulsive cough, or of asthma occur in consequence, or convulsions come on, which terminate life in a few days." (p. 174.)

Such is the course usually run by this affection, but its symptoms differ, when, as is sometimes the case, the hypertrophy is partial, or when the disease assumes the active form, or that in which the walls of the skull, owing to the energy of the process of ossification, do not expand in proportion to the rapid growth of the brain. Its symptoms then are usually those of active cerebral disease, the result of compression of the brain, and its consequent congestion.

In the chapter on chronic hydrocephalus, the diagnosis between that disease and hypertrophy of the brain is stated at great length. The chief differences insisted on by M. Mauthner will perhaps be best seen by throwing them into the following table.

| *Hypertrophy of the Brain.* | *Chronic Hydrocephalus.* |
| --- | --- |
| 1. The posterior part of the skull first presents an unnatural prominence. | 1. The forehead is the first part to present unnatural prominence; the altered direction of the eyes and the very great width of the sutures and fontanelles are likewise characteristic. |
| 2. Children lie horizontally, or throw the head back. | 2. Children lie on the belly, with the head lower than the rest of the body, burying the face in the pillow. |
| 3. Face puffy, eyes inexpressive and staring, mouth half-open. | 3. Countenance withered, having expression of premature old age. |
| 4. Functional disturbance comes on very gradually—not before the period of dentition or weaning—and consists at first in affection of the respiratory apparatus, difficulty of breathing, and attacks of apnœa. | 4. Functional disturbance occurs early and involves the cerebrum from the very beginning. |
| 5. Patient fat and leucophlegmatic. | 5. Patient ill-nourished, subject to rickets and tabes mesenteria. |

The treatment of hypertrophy of the brain necessarily differs according to the circumstances under which it occurs. In that form which is connected with rickets, absorbents and rhubarb with preparations of iron, and a properly regulated diet continued for months, are often very useful. Cold sponging of the surface is frequently of service, but in consequence of the tendency to perspiration about the head care should be taken not to leave it quite bare, but it should be constantly covered with a light cap. In the active form of the disease, whatever might tend to excite the brain must be avoided, while the long-continued use of the iodide of potassium has been found beneficial. Warm pediluvia and the occasional application of small blisters to the back have likewise been of service.

Atrophy of the Brain. In the next chapter the author treats of two conditions which are the direct antitheses of hypertrophy of the brain, namely, atrophy of the brain and the hydrocephaloid disease. The size of the brain has been stated by different writers to be uninfluenced by general emaciation of the body, but though this be to a certain extent true, yet the brain itself loses size and weight in cases of softening or long-continued disease of the stomach and bowels. Dr. Sims has noticed that in old age, in the advanced stages of phthisis, in disease of the stomach, and other affections in which great emaciation occurs, the brain likewise undergoes a partial or general loss of substance. The same wasting of the brain takes place very perceptibly during the rapid atrophy of which children are sometimes the subjects, when the natural turgor of the fontanelles and sutures is sometimes so far diminished that the scalp falls into wrinkles, and the edges of the ununited bones may be made to overlap each other. Children in this condition sometimes live in a state of exhaustion a considerable time; they notice nothing, and neglect their playthings, while the automatic movements of their limbs betray an intelligence no higher than that of brutes. Their face grows old and withered, and every higher expression of the features undergoes a gradual sometimes a total obliteration, while their craving for food and drink becomes insatiable. In older children a somewhat similar retrocession of the intellectual powers occasionally comes on in the course of mesenteric diseases, or of long continued diarrhea. In such cases the weight and size of the brain are found after death much below the average, and there is a considerable interval often occupied by transparent serum between the brain and the parietes of the skull. The pia mater is pale and bloodless, the cerebral substance is soft, anæmic, often infiltrated with serum, and the difference between the gray and white matter is undistinguishable. The convolutions of the organ, and the parts in its interior are but imperfectly developed, and the ventricles are often empty, though sometimes they contain serum, and the characters of atrophy of the brain, then pass gradually into those of chronic hydrocephalus.

The author notices a peculiar condition of the brain which he has sometimes observed in children who have died of marasmus. It consists of a partial induration of the organ, and though often associated with diminution in the size and weight of the brain is sometimes met with independent of any alteration of its volume. He regards it as the result of a state of congestion or inflammation, but the symptoms by which it is attended are very obscure, consisting in convulsions, sopor, and very rapid emaciation. The centrum ovale, and the walls of the lateral ventricles, especially at the anterior or posterior horn, are its most frequent seats, and it is sometimes remarkably evident when it affects the tænia semicircularis. The indurated portion usually has an elongated form, is distinguishable by the gray colour of the cerebral substance, but especially by its cartilaginous hardness. This form of induration of the brain, of which Dr. Mauthner relates three instances has to the best of our knowledge never been noticed by any other writer. It is, therefore, worthy of mention though at present little more than a pathological curiosity.

He next describes that form of atrophy, or rather of deficient development of the brain, which is connected with smallness of the skull, for the most part congenital, constituting microcephalus. Though in point of intellectual endowment such children are on a level with the Cretins, in whom likewise the brain and skull are often inadequately developed, yet there is a wide difference between them in point of physical organization. The body of the former is well developed, and they perform all the functions of animal life perfectly, it is only their brain which seems in fault, while in the latter, all the springs of life seem nearly equally affected by disease, and the ill-formed body, and its ill-executed functions are almost as deplorable as the low intellectual endowment of the child. Children with this congenital smallness of the brain often suffer from early infancy from various convulsive affections, or other indications of diseased innervation; and they either die under the first attack of serious disease, especially if that disease be one of the exanthematous fevers, or they are carried off by the development of tubercular disease.

SOFTENING OF THE BRAIN. In his observations on the hydrocephaloid disease, Dr. Mauthner adds nothing to the well known account of it by Dr. Marshall Hall. We likewise pass over the chapters on chronic hydrocephalus, and on tubercle of the brain, not as by any means valueless, but as containing less that is new or striking than is to be found in some other parts of the work. These are succeeded by a chapter on softening of the brain, a condition which he, like MM. Rilliet and Barthez, considers to be the secondary result of some other disease, not a distinct and independent malady. One anatomical peculiarity which distinguishes softening of the brain in the child from the same process in the adult is that the gray substance is seldom affected, the central white matter being almost always the exclusive seat of the alteration of texture. Dr. Mauthner recognizes the difficulty, indeed we may say the impossibility of determining in many cases where softening of the central parts of the brain has been found to coincide with the accumulation of fluid in the ventricles, which was the primary affection. He does not, however, seem disposed to regard this softening as in all cases of the nature of mere œdema, but thinks it is one of the consequences of a morbid state of the circulating fluid, which tends to produce disintegration of various organs of the body; since this softening is by no means confined to the brain, but affects other parts, particularly the mucous membrane of the intestines. The only symptom of actual destruction of the cerebral fibres, on which he seems disposed to rely is hemiplegia. He confesses even this to be by no means a certain indication.

Convulsions. In the present state of our knowledge of cerebral disease; after all known disorders of the brain have been classified and described as carefully as posssble, the large class of convulsions still remains, the causes of which are so various that there is no single form of cerebral disturbance to which they can be referred. Dr. Mauthner therefore devotes a whole chapter to the subject of convulsions, one too of the most valuable and practically useful in the book. He commences the subject with remarking that convulsions comparatively seldom occur in children as the primary results of disorder of the nervous system, but that they are usually secondary phenomena induced by sympathy with disease in some other part of the organism. This fact is now generally admitted, but Dr. Mauthner is of opinion that the influence of different states of the vascular system in inducing convulsive affections has hardly been sufficiently appreciated. The healthy performance of the functions of the nervous system depends not merely on the normal relations of the peripheral nerves to the brain and spinal cord, but also on the healthy state of its relations to the vascular system.

"Every disturbance of the harmonious reciprocity of action between vessels and nerves, and between the latter and their common centre, must therefore interrupt the equilibrium between the nutrient parts and the parts nourished, and excite a morbid activity of the nervous system, which displays itself very soon in children, through the medium of their irritable muscles. Disturbances of such a kind are extremely frequent at this period of life, owing to the circumstance that the blood has still to pass through many stages of development; that the brain, the centre and regulator of nervous energy, is in a state of constant and rapid growth, and lastly, that the whole vital powers of the child are silently engaged in the task of building up the unfinished organism. Convulsions are often in early life the indications of febrile action, and are a frequent symptom of some local disturbance in the system." (p. 360.)

We somewhat compress the remarks which he goes on to make, in which he endeavours to show that the development of febrile action, and the phenomena which attend it, depending on a high degree of vigour in the vascular system; the absence of this power in the vascular system in childhood accounts for the absence of the symptoms which characterizes pyrexia. That very tendency to venous congestion, which is so strikingly marked in childhood, increases the excitability of the nervous system, and produces a liability to convulsive affections. Of the truth of this statement the numerous hypochondriacal and hysterical affections that occur in the adult are ample evidence, as are the cramps and spasmodic pains which attend deranged menstruation and hemorrhoidal affections.

Now it is very probable that there may be in the remarks just quoted rather more of the transcendentalism of the Vienna School than will suit all our readers. Truth, however, forms the basis of Dr. Mauthner's remarks, and perhaps we should not find it easy to express his meaning better. He next glances at the different organs whose disorders are often attended with convulsions, among which the brain and its diseases hold the foremost place. He notices the frequent occurrence of convulsions as the fatal termination of various affections draws nigh, when they indicate that death has begun to assail the centres of life. His next remark is one that we have not seen before in print, but we can confirm it from our own observation, except that we do not think the cessation of thoracic symptoms is quite so complete as he here represents it.

"I have," says he, "often observed in cases of extensive hepatization or tuberculization of the lungs, during the course of which the brain was perfectly unaffected, that the children a few days before death lost all chest symptoms, that the cough and orthopnea seemed to have entirely vanished, their appetite returned, and they seemed cheerful, when convulsions suddenly came on, followed in a few hours by death." (p 362.)

Equally correct is the following statement:

"Another tolerably frequent phenomenon is this, that in those cases of cerebral disease which were marked at their commencement and during their course by violent convulsions, the act of dying is generally quite tranquil, owing to the paralysis of the brain having suspended its influence over the muscular system." (p. 363.)

Before he passes to the consideration of the treatment, he introduces one very important caution, which we must extract:

"Inasmuch as convulsions are a frequent attendant on disease of the brain, it is certainly very natural to turn one's attention first to the nervous centre. It often happens, however, if much care be not taken to investigate a case thoroughly, that leeches and cold applications to the head are hastily ordered, and calomel given; when the presence of pneumonia is afterwards detected, or some cause of gastric disturbance found to exist, without due attention to which no permanent amendment can result from any treatment. Inflammations of the chest are particularly apt to lead into this kind of error. Their real symptoms are masked by convulsive seizures, the medical attendant fancies on the first day that the case is one of inflammation of the brain, on the next day he thinks it must be pneumonia, and thus the uncertain diagnosis leads to vacillating treatment, and much mischief is the result." (p. 364.)

We do not enter into any minute account of the treatment suggested by Dr. Mauthner in the different forms of convulsive attacks. The judicious employment of known remedies, not the eager hunting after new medicines, has long been the characteristic of the sound physician. The cases appended to this chapter afford the best possible illustration of the various forms which these affections assume, and the various treatments they require.

The last eighty pages contain an account of diseases of the spinal cord in children. They contain much that is deserving of notice, but we have already exceeded our limits, and must take leave of Dr. Mauthner, heartily recommending his book as the most valuable contribution to practical medicine which has appeared for many years from the Vienna school.

---

